# Meat Consumption and Meat Cooking Practices in Essential Tremor: A Population-Based Study in the Faroe Islands

**DOI:** 10.5334/tohm.236

**Published:** 2020-08-14

**Authors:** Monica Ferrer, Eina H. Eliasen, Maria Skaalum Petersen, Wendy Jiang, Wei Zheng, Elan D. Louis

**Affiliations:** 1 Department of Neurology, Yale School of Medicine, Yale University, New Haven, CT, US; 2 Department of Occupational Medicine and Public Health, The Faroese Hospital System, Tórshavn, FO; 3 Centre for Health Science, Faculty of Health Sciences, University of the Faroe Islands, Tórshavn, FO; 4 Purdue University School of Health Sciences, West Lafayette, IN, US; 5 Department of Neurology and Neurotherapeutics, University of Texas Southwestern, Dallas, TX, US

**Keywords:** environmental epidemiology, population-based design, essential tremor, diet, neurotoxin, harmane

## Abstract

**Background:**

Elevated tissue levels of the tremor-producing neurotoxin, harmane, have been detected in patients with essential tremor (ET) in the USA and Spain. Recently, a study in the Faroe Islands similarly noted an elevation in blood harmane concentrations in probable and definite ET cases. The underlying mechanism is not understood. Possible mechanisms include increased dietary consumption (esp. through cooked meats), impaired metabolism, or increased endogenous production of harmane. To investigate this issue further, we conducted a population-based study in the Faroe Islands to examine meat consumption and meat cooking practices in ET cases and controls.

**Methods:**

1,328 Faroese adults were screened for tremor and 27 ET cases were identified. Meat consumption and meat cooking practices were compared to 200 controls. Detailed data were collected via questionnaires regarding current meat consumption for 14 meat types and meat cooking doneness for 8 meat types. Data were also available on blood harmane concentrations.

**Results:**

Current meat consumption was similar in ET cases and controls in 12 out of 14 meat types, with no differences observed after a Bonferroni correction in any meat type; no difference was observed when stratified by gender. No difference was observed in meat doneness between ET cases and controls. Blood harmane concentrations were not correlated with dietary data.

**Discussion:**

This is the first population-based study of harmane-linked dietary factors in ET. The study suggests the observed difference in blood harmane in ET is not driven by dietary differences and is likely due to other mechanisms (e.g., impaired metabolism).

## Introduction

Essential tremor (ET) is among the most prevalent neurological diseases, yet potential modifiable environmental factors contributing to its etiology are poorly understood. Past studies have identified exposure to β-carboline alkaloids (BCAs), especially harmane, as a possible link to ET [1,2,3].

Indeed, BCA-induced tremor has been used as the major animal model for ET, as the induced tremor shares principal clinical features with ET, drug-response characteristics, and some of the underlying brain changes [1]. Interestingly, blood and brain concentrations of harmane have been shown to be elevated in patients with ET compared to controls [2,4,5,6]. Furthermore, a magnetic resonance spectroscopic imaging study showed that blood harmane concentration correlated with a marker of cerebellar neuronal damage [7].

The basis for the elevated blood and brain harmane in ET cases is unknown. While harmane is produced endogenously by the body, exogenous sources (i.e., exposure through the diet) is considered the major source [8]. In fact, harmane is the most abundant of all dietary BCAs, and is found naturally in a variety of plant species and meats, and concentrations may increase exponentially during food heating processes [1,8,9]. Meats and especially meats with long cooking times and high doneness levels have particularly high concentrations [10].

The detection of a potential modifiable factor, like meat consumption, would be critical in ET, a disease that currently has no cure and poor treatment options. Weakly supporting the hypothesis that higher harmane levels in ET cases are due to greater dietary exposure, one study observed slightly greater meat consumption in males with ET than in control males, however these results did not extend to females and, furthermore, meat cooking practices (i.e., doneness levels) were similar in cases and controls [11].

Our study builds on this finding and examines the association between meat consumption and meat cooking practices and ET in a population-based study in the Faroe Islands. The Faroe Islands were selected for the studies given their high prevalence of neurological disorders and special dietary habits rich in fish, seabirds, mutton, pilot whale meat and blubber (i.e., meats) [12]. The prevalence of several movement disorders, such as Parkinson’s disease (PD) and dystonia, has also been noted to be high within this population [13,14,15,16]. Additionally, its genetically homogenous, highly delimited and well-defined population, make the Faroe Islands a well-suited site to conduct epidemiological studies. Recently, a study in the Faroe Islands noted a mild elevation in blood harmane concentrations in probable and definite ET cases [3], further facilitating and prompting the current analyses. The underlying mechanism for this elevation is not understood, and in these analyses, we explored dietary ones.

## Methods

### Study Population

Participants were identified using a two-phase, population-based study design described previously [3,16,17,18]. All study procedures were approved by the local Ethical Review Committee of the Faroe Islands and the Institutional Review Board at Yale University, with participation on a voluntary basis and signed informed consent obtained from each enrollee. The names and addresses of 4,798 individuals aged ≥40 years were obtained from the Faroese Population Registry from a sample of 24,154 individuals aged ≥40 years living in the Faroe Islands. These 4,798 individuals were selected based on six randomly selected birthdates [17]. From this group 3,000 individuals were selected to comprise the screening group. All individuals ≥70 years (n = 1,155), were automatically selected into the screening group. The remaining 1,845 were selected by random sampling using IBM’s Statistical Package for the Social Sciences (SPSS).

### First Phase Screening

During the first screening phase 3,000 individuals received an invitation letter to participate in a study of lifestyle, diet, and neurological conditions [17]. The invitation letter included a screening package that contained questionnaires, a request for hand-drawn spirals, and a return stamped envelope. The questionnaires included demographic and clinical questions, seven screening questions for tremor, and instructions on how to draw two Archimedes spirals with each hand [17]. A total of 1,334 (44.5%) individuals returned the completed screening package [17].

Tremor on each spiral was rated by senior movement disorders neurologist (E.D.L) using an ordinal clinical rating scale (0–3.0). Based on data from the questionnaire and spiral scores, participants were stratified into 4 groups: (1) high likelihood of having ET, (2) intermediate likelihood of having ET, (3) low-intermediate likelihood of having ET, and (4) low likelihood of having ET [17].

### Second Phase

A subset of 282 individuals who returned the spirals and questionnaires were invited to participate in an in-person clinical evaluation [17]. All 85 individuals in group 1 (high likelihood of having ET) were invited. A randomly selected sample of 97 individuals from group 2, 32 individuals from group 3, and 68 individuals from group 4 were invited. A total of 227 (80.5%) individuals accepted and completed the in-person clinical evaluation, while 54 declined participation and one person died. The participation rate was 78% (group 1), 85% (group 2), 84% (group 3), and 76% (group 4) [17].

The in-person clinical evaluation [17] was conducted by a trained nurse (E.H.E) and included (1) anthropometric measures (body weight in kg and height in cm); (2) a questionnaire encompassing data on demographics, education, medication usage, smoking habits, ethanol intake, fasting status, and family history of ET or tremor, (3) detailed data on current meat consumption and cooking practices, (4) a detailed videotaped tremor examination, and (5) phlebotomy for blood harmane. Pack years for each subject was calculated by multiplying the number of packs of cigarettes smoked per day by the number of years the subject has smoked. Body mass index (BMI) was calculated by dividing weight in kilograms by height in meters squared.

### Meat Consumption and Cooking Practices

The detailed data on current (i.e., during the past year) meat consumption was collected using a modified version of the Lawrence Livermore National Laboratory Meat Questionnaire (LLNL) [19,20], which was further modified to include three extra meat types consumed in the Faroe Islands (whale, sea bird and lamb). The questionnaire included questions related to consumption of 14 specific meats: (1) frequency of consumption (coded into nine categories ranging from never to every day) and (2) portion size (number of standard sized portions as represented in photographs presented to participants) [11]. As described [11], a summary measure of total quantity of meat consumption was generated. Total quantity of meat consumption was calculated by multiplying each meat frequency (per day) by the portion size of that meat (in grams) to generate the number of grams per day for each meat type and this was summed to give total meat consumption (grams/day).

Meat doneness for 8 meat-types was assessed utilizing self-reported cooking doneness (1 = rare or medium to 4 = extra well done). Additionally, doneness for 6 meat-types was reported through a series of photographs demonstrating four different levels of meat doneness (1 = rare to 4 = charred) [11]. Two summary measures, total current self-reported cooking doneness (i.e., the doneness levels for all 8 meat types combined) and total current self-reported photo-based cooking doneness (i.e., the doneness levels for all 6 meat types combined), was calculated for each participant [11].

### Videotaped Tremor Examination

The videotaped tremor examination included (1) an ET specific metric comprising one test for postural tremor and five for kinetic tremor (e.g., pouring, drinking) performed with each arm (12 tests total, with total tremor score = 0–36) [21], (2) the motor portion of the Unified Parkinson’s Disease Rating Scale (UPDRS) excluding an assessment of rigidity [22], and (3) an assessment of dystonia [16].

### ET Diagnosis

ET diagnoses (definite, probable, possible as described in previous studies) were assigned by E.D.L by review of questionnaire data, the videotaped neurological examination, and based on published diagnostic criteria (moderate or greater amplitude kinetic tremor during 3 or more activities or a head tremor in the absence of PD or another known cause such as medication-induced tremor or tremor from hyperthyroidism) [17,23]. As reported previously, a diagnosis of ET was assigned to 27 of 227 individuals [17].

### Measurement of Blood Harmane Concentration

After phlebotomy, blood harmane was quantified, blinded to clinical information, by a well-established high performance liquid chromatography method (Dr. Zheng, Purdue University) used in previous studies [2,4].

### Statistical Analyses

Statistical analyses were performed using SPSS version 25.0. Differences between the ET and non-ET group were tested with Student’s t-test for continuous variables or chi square test for categorical variables. Mann-Whitney tests were used when continuous variables were not normally distributed.

Neither total quantity of meat nor quantity of meat types consumed were normally distributed (Kolmogorov-Smirnov test); hence, nonparametric tests were used to compare quantity across diagnostic groups, and median values were also reported. Meat cooking practices were evaluated for eight meat types (chicken, hamburger/ground beef, beef, bacon, pork, fish, lamb, seabird) utilizing self-reported cooking doneness. A Mann-Whitney test was used for case-control comparisons for each meat type doneness as well as for the total current self-reported cooking doneness (i.e. the doneness level for all 8 meat types combined for each participant). Six meat types’ (chicken, hamburger, steak, bacon, pork, and fish) cooking doneness was further examined using meat doneness scores as represented by photos. Total current self-reported photo-based cooking doneness (i.e., the doneness levels for all 6 meat types combined), was calculated for each participant and compared between cases and controls (Mann-Whitney test). For all analyses, given the number of comparisons, a Bonferroni correction was performed.

Three logistic regression analyses (outcome = ET vs. control) were performed, yielding odds ratios (OR) and 95% confidence intervals (CI). The independent variable was total quantity of meat consumed in the first analysis, total current self-reported cooking doneness in the second analysis, and total current self-reported photo-based cooking doneness in the last analysis. The choice of covariates in adjusted analysis was based on current case-control differences and results of published analyses [2,11,24]. These included age, gender, educational level, cups of coffee on day of examination, body mass index (BMI), current cigarette smoking status, and cigarette pack years.

Correlations between harmane concentrations and dietary and demographic data were examined separately in ET cases and controls. Continuous or ordinal variables were evaluated using a Spearman’s correlation (i.e., total quantity of meat consumption, total current self-reported cooking doneness, total current self-reported photo-based cooking doneness, age, BMI, number of years since most recent hospitalization, cigarette pack years, cups of coffee consumed on day of examination, and educational level). Nominal values were evaluated using a Mann Whitney test (i.e., current cigarette smoking status, gender, and marital status).

### Study Power

The study sample size was fixed as there were 27 ET cases and 200 controls. For *post-hoc* power calculations, data from controls were used and alpha = 0.05 with two-sided testing assumed. For total meat consumption (grams/day), the sample was powered at 82.1% to detect a 37% increase in meat consumption between ET cases and controls. For total current self-reported cooking doneness, the study had a power of 81.3% to detect a 10% increase in meat doneness in ET cases vs. controls. For total current self-reported photo-based cooking doneness, the power was 82% to detect a 13% increase in meat doneness in ET cases vs. controls.

## Results

### Demographic Characteristics of Study Population

A total of 227 subjects participated in the in-person clinical evaluation. There were 27 ET cases and 200 controls. Cases and controls were similar in terms of demographic and clinical characteristics (Table 1, all *p* values > 0.05).

**Table 1:** Demographic characteristics of ET cases and controls.

	ET cases	Controls

	(n = 27)	(n = 200)

Age, years	66.5 ± 11.7	62.2 ± 12.3
Gender		
Male	17 (63.0%)	107 (53.5%)
Female	10 (37.0%)	93 (46.5%)
Level of education		
Less than high school	8 (29.6%)	54 (27.0%)
High school	2 (7.4%)	7 (3.5%)
Less than college	8 (29.6%)	63 (31.5%)
College graduate	5 (18.5%)	58 (29.0%)
Master or doctorate degree	2 (7.4%)	14 (7.0%)
Missing	2 (7.4%)	4 (2.0%)
Current cigarette smoker	9 (33.3%)	40 (20.0%)
Smokers’ pack years	18.8 ± 13.9	21.5 ± 16.4
Marital status		
Married, co-habiting or remarried	13 (48.1%)	138 (69.0%)
Widowed	6 (22.2%)	22 (11.0%)
Never married	5 (18.5%)	22 (11.0%)
Divorced or separated	2 (7.4%)	14 (7.0%)
Other	1 (3.7%)	4 (2.0%)
Years since last hospitalization	7.8 ± 7.8	11.6 ± 13.6
Body mass index	28.0 ± 3.8	28.4 ± 4.6
Coffee cups consumed on examination day	2.2 ± 2.4	1.8 ± 2.4

Values represent mean ± standard deviation or number (percentage).

### Quantity of Meat Consumed in ET Cases and Controls

Total quantity of meat consumption was similar in cases and controls (mean ± SD): 120.0 ± 110.7 g/day vs. 113.6 ± 71.2 g/day (median values 89.0 vs. 95.8; *p* = 0.90). Total quantity of meat consumption was also similar when stratified by gender: 133.2 ± 134.6 g/day for male cases vs. 137.7 ± 80.3 g/day for male controls (median values 92.3 vs. 116.0; *p* = 0.23) and 97.7 ± 48.2 g/day for female cases vs. 85.8 ± 45.6 g/day for female controls (median values 87.6 vs. 74.1; *p* = 0.47). In a multivariate logistic regression analysis, adjusting for age, gender, educational level, cups of coffee on day of examination, BMI, current cigarette smoking, and cigarette pack years, the total quantity of meat consumption did not differ in cases and controls (OR = 1.00, 95% CI = 1.00–1.01, *p* = 0.24).

When different meat types where evaluated separately, 12 out of 14 meat types were consumed in similar quantities per day, exception for pork chops and bacon (Table 2). After a Bonferroni correction (α_altered_ = 0.05/14 = 0.0036), these two differences were no longer significant.

**Table 2:** Quantity of meat consumption for different meat types.

	ET cases	Controls	*p* Value*

	(mean ± SD g/day)	(mean ± SD g/day)	

Pan fried chicken	0.8 ± 1.6	2.7 ± 8.6	0.12
Chicken grilled/fried/broiled in oven or BBQ**	10.2 ± 15.9	8.8 ± 9.7	0.50
Hamburger from ground beef	10.5 ± 9.5	10.4 ± 11.3	0.92
Steak	2.7 ± 3.1	5.5 ± 7.8	0.12
Joint of beef (beef ribs, roast rump steak, etc.)	6.5 ± 6.5	6.9 ± 9.3	0.87
Pork roast, rib roast, pork rib	10.2 ± 24.2	5.3 ± 6.3	0.48
Pork chops	8.1 ± 12.3	4.2 ± 5.4	**0.03**
Bacon	12.9 ± 24.4	7.4 ± 13.9	**0.01**
Pan fried fish	18.3 ± 17.0	15.2 ± 13.0	0.37
Fish grilled/fried/broiled in oven or BBQ	5.0 ± 7.6	5.4 ± 10.3	0.78
Mixed meat dishes	7.5 ± 7.5	13.7 ± 17.7	0.12
Lamb broiled/grilled in oven or BBQ	14.4 ± 16.2	13.9 ± 11.8	0.62
Sea bird grilled/broiled in oven or BBQ	1.6 ± 2.0	2.2 ± 3.4	0.21
Whale meat	3.1 ± 1.9	4.0 ± 5.1	0.40

* Mann-Whitney test; ** BBQ = Barbecue; Bold font = statistically significant.

As discussed previously [3], median blood harmane concentration was observed to be 2.7 times higher in definite ET (4.13 ng/ml) and 1.5 times higher in probable ET (2.28 ng/ml) than controls within this population. When stratified by diagnostic groups to compare definite and probable ET cases (n = 16) to controls we did not observe any significant difference between total quantity of meat consumption: 123.9 ± 142.8 g/day vs. 113.6 ± 71.2 g/day (median values 85.3 vs. 95.8; *p* = 0.53).

### Meat Cooking Practices in ET Cases and Controls

Meat cooking practices were evaluated utilizing total current self-reported cooking doneness as well as total current self-reported photo-based cooking doneness. First, meat cooking doneness for eight different meat types was evaluated. The total current self-reported cooking doneness was similar in ET cases and controls (mean ± SD): 14.5 ± 2.5 vs. 14.9 ± 3.0 (median values 14.0 vs. 15.0; *p* = 0.56). Second, six meat types were further analyzed using the selected photos representing meat doneness. Meat cooking doneness represented in photos for the six different meat types was also similar between ET cases and controls. The total current self-reported photo-based cooking doneness was similar in ET cases and controls: 11.8 ± 2.6 vs. 11.7 ± 2.6 (median values 12.0 vs. 12.0; *p* = 0.85).

When stratified by gender, total current self-reported cooking doneness and total current self-reported photo-based cooking doneness were also similar between ET cases and controls. Total current self-reported cooking doneness for males ET cases was 14.2 ± 2.7 vs. 15.0 ± 2.8 for male controls (median values 14.0 vs. 15.0; *p* = 0.41), and 15.0 ± 2.2 for female cases vs. 14.9 ± 3.1 for female controls (median values 14.5 vs. 15.0; *p* = 0.93). Total current self-reported photo-based cooking doneness for males ET cases was 11.3 ± 2.6 vs. 11.3 ± 2.5 for male controls (median values 11.0 vs. 11.0; *p* = 0.93), and 12.7 ± 2.4 for female cases vs. 12.1 ± 2.6 for female controls (median values 13.5 vs. 12.0; *p* = 0.51).

In two multivariate logistic regression analyses, adjusting for age, gender, educational level, cups of coffee on day of examination, BMI, current smoking, and cigarette pack years, total current self-reported cooking doneness and total current self-reported photo-based cooking doneness did not differ in cases and controls (OR = 0.96, 95% CI = 0.81–1.12, *p* = 0.58 and OR = 1.00, 95% CI = 0.84–1.19, *p* = 0.97, respectively).

When stratified by diagnostic group, total current self-reported cooking doneness was similar in cases of definite and probable ET compared to controls (mean ± SD: 14.6 ± 2.7 vs. 14.9 ± 2.9; median values 14.5 vs. 15.0; *p* = 0.78). Total current self-reported photo-based cooking doneness was also similar in cases of definite ET and probable ET compared to controls (12.1 ± 2.6 vs. 11.7 ± 2.6; median values 12.5 vs. 12.0; *p* = 0.52).

### Correlates of Blood Harmane Concentration in ET Cases and Controls

In controls, blood harmane concentration was not found to be associated with total current meat consumption (Spearman’s r, r_s_ = 0.04, *p* = 0.57), total current self-reported cooking doneness (r_s_ = 0.04, *p* = 0.60), or total current self-reported photo-based cooking doneness (r_s_ = 0.02, *p* = 0.77). Increased harmane concentration was only found to be slightly associated with an increased BMI (r_s_ = 0.15, *p* = 0.04) and increased age (r_s_ = 0.15, *p* = 0.05). Blood harmane concentration was not associated with other potential confounders, including gender, level of education, marital status, years since last hospitalization, pack years, current smoking, and cups of coffee consumed on day of examination (all p values > 0.05).

In ET cases, blood harmane concentration was similarly not found to be associated with total current meat consumption (r_s_ = –0.17, *p* = 0.39), total current self-reported cooking doneness (r_s_ = –0.23, *p* = 0.25), or total current self-reported photo-based cooking doneness (r_s_ = 0.16, *p* = 0.42). However, in ET cases increased harmane concentration was associated with increased pack years (r_s_ = 0.40, *p* = 0.04) and current smoking (*p =* 0.007). Blood harmane concentration was not associated with other potential confounders, including age, gender, level of education, marital status, years since last hospitalization, cups of coffee consumed on day of examination, and BMI (all p values > 0.05).

## Discussion

The mechanism that underlies the observed elevation in tissue levels of harmane in ET is unclear. We had hypothesized that the increased harmane concentration could be due to exogenous consumption of harmane through dietary protein. However, we did not detect any significant difference between current animal protein consumption in ET cases vs. controls. Although, ET cases reported significantly higher consumption of 2 out of 14 meat types (pork chops and bacon), after a Bonferroni correction (α_altered_ = 0.05/14 = 0.0036) the differences were no longer significant. In a study in New York, the Willet Semi-Quantitative Food-Frequency Questionnaire was used to measure food frequency of 61 nutrients [24]. In that study, total daily animal protein consumption was similar in ET cases and controls (50.2 ± 19.6 vs. 49.4 ± 19.1 g/day, p = 0.74) [24]. However, in a follow-up study in New York [11], the LLNL Meat Questionnaire was utilized as it provided more detailed information of consumption of 14 specific meat types. In that study [11], it was noted that total meat consumption was greater in males with ET vs. males without ET (135.3 ± 71.1 vs. 110.6 ± 80.4 g/day, p = 0.03). The observation was questionable, given the difference was only restricted to males and was not found in females. The study was not population-based, as is the current one we now report. Our current study, which used the more detailed questionnaire (LLNL) and was population-based, did not appreciate any differences between meat consumption between ET cases and controls.

Additionally, we did not note any difference in meat cooking practices and level of meat doneness between ET cases and controls, further suggesting that the case-control difference we observed in harmane concentrations are not driven by dietary differences. These data support previous studies in New York that did not note any differences between cooking practices [11,24]. Furthermore, we did not note any correlation between dietary data and harmane concentrations in ET cases or controls, suggesting that there may be factors besides dietary intake that are responsible for the elevated blood harmane in patients with ET.

The basis for the elevated blood harmane in ET cases is unknown, but our results in conjunction to previous studies support the idea that it could be the result of a genetically driven reduction in harmane metabolism. BCAs may undergo two different metabolic pathways. After oral administration, BCAs can pass the blood-brain barrier and get bioactivated by N-methyltransferases (NMTs) into N-methyl-B-carbolinium cations (2,9-diMe-BC+). These BCA cations can then inhibit mitochondrial complex I, increase reactive oxygen species (ROS) production and induce cell apoptosis [9,25]. Alternatively, BCAs can bypass the formation of these neurotoxic cations and instead be metabolized by cytochrome P-450 enzymes to hydroxylated derivatives in a detoxification process [9,26]. Harmane is metabolized by P-450 to give the metabolite harmine [25,27]. Supporting a genetic/metabolic process, previous studies have noted an increase in harmane in familial ET vs. sporadic ET [5]. Furthermore, the metabolism of harmane to harmine by the liver cytochrome P-450 enzyme was studied in ET cases [5]. In this study it was shown that harmane/harmine ratio was highest in familial ET, intermediate in sporadic ET and lowest in the control. Individuals with slow hydroxylation capacity may have increased susceptibility to disease due to the accumulation of toxic metabolites. Future studies should assess liver function, variability in cytochrome P-450, and bioactivation to BCA cations to explore whether the metabolism of harmane differs in ET cases and controls.

This study should be interpreted within the context of several limitations. We did not assess fasting levels of harmane; however, prior data show blood harmane is not dependent on time of last food consumption and that there is no association between time elapsed from last food ingestion and blood harmane concentrations [5]. Also, we assessed subjects’ current dietary intake and blood harmane concentrations, not their premorbid state. We thus cannot comment on the link between premorbid dietary habits on the development of ET. Furthermore, while previous studies have shown harmane concentrations are highest in familial ET [5], we were not able to stratify ET cases by family history given that nearly all of the ET cases had not previously been diagnosed nor had they explored family history for tremor disorders.

This study has considerable strengths. First, this is the first population-based study of dietary meat consumption and harmane concentration in ET, with all prior studies largely or exclusively sampling cases from clinics [2,4]. Second, our study utilized the more detailed LLNL Meat Questionnaire allowing us to collect detailed information of specific meat types. Finally, our study expands the investigation of dietary risk factors to a new population, increasing the diversity of investigation and strengthening the links between this neurotoxin and ET.

In summary, we were not able to detect any robust dietary and cooking difference between ET cases and controls or a correlation between blood harmane concentrations and meat consumptions. These data lend further credence to the notion that non-dietary factors (e.g., metabolic and genetic differences) underlie the case-control difference in blood harmane concentrations observed in this or previous studies [11,24]. The link between harmane and ET has been established now in three different populations – the USA [2], Spain [4], and the Faroe Islands [3]. Future research must now focus on examining the mechanism of this elevation.
